# Graphene Oxide-Modified Hydroxy-Terminated Polybutadiene Liner for Suppressing Plasticizer Migration: Molecular Dynamics Simulation and Experimental Study

**DOI:** 10.3390/polym18202490

**Published:** 2026-10-12

**Authors:** Junqiang Feng, Wanru Cheng, Liang Xu, Yuhan Meng, Chenxing Liu, Tao Ding, Yubing Hu, Jun Zhou, Fuyao Chen, Zhehong Lu

**Affiliations:** 1 College of Chemistry and Molecular Sciences, Henan University, Kaifeng 475004, China; 2 Shanxi Jiangyang Chemical Co., Ltd., Taiyuan 030041, China; 3 Queen’s Business School, Queen’s University Belfast, Belfast BT9 5AH, UK; 4 School of Chemistry and Chemical Engineering, Nanjing University of Science and Technology, Nanjing 210094, China; hyb@njust.edu.cn; 5 School of Physics and Materials Engineering, Hefei Normal University, Hefei 230061, China; 6 Tongxiang Research Institute, Zhejiang Sci-Tech University, Jiaxing 314000, China

**Keywords:** liner, graphene oxide, anti-plasticizer migration, molecular dynamics simulation

## Abstract

In solid rocket motors, the liner serves as a critical interfacial material between the propellant and insulation, and its resistance to plasticizer migration directly affects the long-term structural stability of the propellant grain. In this study, hydroxyl-terminated polybutadiene (HTPB) was used as the matrix and isophorone diisocyanate (IPDI) as the curing agent to prepare composite liners with varying graphene oxide (GO) contents (0, 0.1, 0.3, and 0.5 wt%). The ability of the liners to suppress the migration of dioctyl sebacate (DOS) was systematically investigated. Molecular dynamics (MD) simulations were also conducted by constructing two amorphous cell models, namely DOS/HTPB/IPDI and GO/DOS/HTPB/IPDI, to calculate binding energy and migration coefficient and elucidate the role of GO at the molecular level. Immersion experiments revealed that the incorporation of GO significantly lowered the migration rate of DOS in the liner, with the optimal anti-migration performance achieved at a GO content of 0.3 wt%, which exhibited the lowest equilibrium migration concentration. MD showed that the incorporation of GO reduced the binding energy between DOS and the matrix from 290.998 to 229.264 kcal/mol, while the migration coefficient of DOS decreased from 6.52 × 10^−11^ m^2^/s to 5.52 × 10^−11^ m^2^/s, corresponding to a reduction of approximately 15.3%. The combined experimental and simulation results indicate that an appropriate amount of GO forms a two-dimensional physical barrier within the HTPB/IPDI matrix, effectively extending the diffusion pathway of DOS molecules and weakening their affinity with the matrix, thereby significantly enhancing the anti-migration performance of the liner. This work provides both experimental and molecular-level insights for the development of high-performance liner materials with enhanced resistance to plasticizer migration.

## 1. Introduction

Solid rocket motors (SRMs) have been widely employed in strategic and tactical missiles as well as aerospace vehicles owing to their simple structure, rapid response, and high operational reliability. The safety and stability of an SRM are therefore of critical importance to the overall reliability of the platform on which it operates. In an SRM, the propellant grain is bonded to the motor case through the liner and insulation, forming three critical interfaces, namely, the propellant–liner, liner–insulation, and insulation–case interfaces [1]. Since storage accounts for most of the service life of an SRM, the probability of structural degradation and interfacial failure increases significantly during long-term storage. In particular, the propellant grain and bonded interfaces are considered to be among the critical failure-prone regions of large SRMs during prolonged storage [2]. If the insulation becomes poorly bonded to the propellant surface, high-pressure combustion gases may penetrate into the debonded region during motor ignition and pressurization. The resulting deformation of the debonded cavity and the associated increase in the effective burning area may cause uncontrolled chamber pressure and, in severe cases, catastrophic motor failure. Therefore, as a critical bonding and transition layer between the propellant and insulation, the liner plays an essential role in maintaining the structural integrity and operational safety of SRMs. Specifically, the liner performs three primary functions. First, it provides strong adhesion between the propellant grain and insulation, preventing interfacial debonding during curing, storage, transportation, and motor operation and thereby maintaining the structural integrity of the grain [3]. Second, it contributes to protecting the inner surface of the motor case from erosion by high-temperature combustion gases [4]. Third, the liner acts as a barrier against the migration of low-molecular-weight components, such as plasticizers, curing agents, and moisture, from the propellant toward the insulation, thereby mitigating interfacial degradation caused by component migration [5].

With the rapid development of solid propulsion technology, increasingly stringent requirements have been imposed on liner materials, particularly in terms of compatibility, mechanical properties, adhesion, and resistance to molecular migration. Compatibility is a key parameter for evaluating the stability and service reliability of propellant systems. The compatibility between the liner and propellant essentially requires coordinated chemical and physical interactions between the two materials. Chemical compatibility requires that no detrimental chemical reactions occur between the liner and propellant during contact, which could otherwise interfere with the curing process or weaken the interfacial adhesion. Physical compatibility, meanwhile, is closely associated with the resistance of the liner to the migration of low-molecular-weight components, including nitroglycerin, burning-rate catalysts, and curing agents, from the propellant. Poor compatibility between the propellant and liner can result in pronounced migration of these components, leading to deterioration of the mechanical properties and structural integrity of the liner and propellant. In addition, undesirable reactions between different components may occur. If the heat generated by such reactions cannot be dissipated efficiently, heat accumulation within the propellant grain may progressively compromise its thermal stability and, under critical conditions, may result in combustion or even explosion, posing a serious safety hazard [6].

In addition to compatibility, the liner must possess sufficient tensile strength, elongation at break, and tear resistance to withstand environmental and mechanical stresses during transportation, storage, and operation. These properties are essential for preventing cracking or debonding of the propellant system under external stresses. For solid rocket motors, the quality and durability of interfacial bonding are among the key factors limiting the service life of the motor [7]. Accordingly, the liner must maintain adequate adhesion under long-term storage and harsh environmental conditions to ensure reliable structural integrity and prevent aging-induced interfacial failure. More importantly, solid propellants contain various mobile low-molecular-weight components, among which plasticizers are introduced primarily to improve mechanical flexibility and mechanical properties while also contributing to the energy performance of the propellant. During long-term storage, however, plasticizers can gradually migrate from the propellant into the liner and insulation. Typical plasticizers, such as dioctyl sebacate (DOS) and dibutyl phthalate (DBP), can accumulate at the propellant–liner interface, causing swelling and softening of the liner while simultaneously inducing hardening and shrinkage of the propellant. These changes can generate considerable local stresses and strains at the interface, resulting in weakened interfacial adhesion and, ultimately, interfacial debonding or structural failure [8]. Therefore, developing liner materials with enhanced resistance to plasticizer migration is of considerable importance for improving the long-term reliability and safety of solid rocket motors.

The hydroxyl-terminated polybutadiene (HTPB)/isocyanate curing system has been widely employed in liners for HTPB-based composite solid propellants and high-energy composite propellants owing to its low cost, excellent mechanical and processing properties, good compatibility with various fillers, as well as favorable hydrolytic stability and aging resistance [9]. HTPB is a telechelic liquid rubber that emerged in the 1960s and is characterized by low viscosity, good transparency, and a relatively low glass transition temperature. More importantly, the polymer chains contain reactive primary hydroxyl groups at both termini, which can undergo crosslinking reactions with isocyanate curing agents at room or elevated temperatures, forming polyurethane elastomers with three-dimensional network structures. Such polyurethane elastomers exhibit high strength, hardness, wear resistance, and excellent low-temperature performance, making them attractive for a wide range of applications [10]. Among the various isocyanate curing agents used for HTPB-based liners, isophorone diisocyanate (IPDI) is one of the most commonly employed. During the curing reaction between HTPB and IPDI, the isocyanate group attached to the cycloalkane moiety exhibits relatively high reactivity and preferentially participates in the reaction, whereas the isocyanate group connected to the methylene group shows relatively lower reactivity due to steric hindrance and reacts at a later stage. This sequential reaction process is conducive to the formation of a relatively uniform three-dimensional crosslinked network, thereby providing the liner with favorable mechanical properties and aging resistance.

Plasticizer migration is a complex physicochemical process governed by multiple factors rather than by a single factor. In recent years, concentration-gradient diffusion, polarity interactions, and chemical-potential gradients have been widely employed to explain the driving forces underlying plasticizer migration [11,12,13,14]. The migration rate and extent of low-molecular-weight components such as plasticizers are strongly dependent on both the composition of the propellant and the physicochemical characteristics of the liner. The propellant composition and plasticizer content directly affect the concentration distribution and migration tendency of plasticizers. In general, a higher plasticizer content produces a larger concentration gradient between the propellant and liner, thereby accelerating the migration process. The intrinsic physicochemical properties of the liner are also critical to its resistance to plasticizer migration. In general, polymers with relatively low polarity exhibit poor compatibility with highly polar nitrate-ester plasticizers and are therefore less susceptible to swelling. Highly crosslinked polymer networks can form dense three-dimensional structures that restrict chain mobility and reduce the available free volume, thereby hindering the penetration of small molecules [15]. In addition, highly crystalline regions can function as physical crosslinking domains and provide more ordered barriers against molecular transport [16].

Based on the above mechanisms and influencing factors, several strategies have been developed to counter plasticizer migration, including optimization of the liner formulation and incorporation of nanofillers [17]. Graphene is a two-dimensional carbon nanomaterial composed of carbon atoms covalently bonded through *sp^2^* hybridization. It possesses outstanding mechanical, electrical, and thermal properties, while its diverse functionalization strategies enable tunable properties and favorable interfacial interactions with polymer matrices, attracting considerable attention in polymer nanocomposites [18]. The theoretical thickness of monolayer graphene is only approximately 0.34 nm, while the geometric pore size of its carbon rings is approximately 0.064 nm, which is substantially smaller than the kinetic diameters of most gas molecules. Consequently, graphene exhibits exceptional resistance to molecular permeation. Graphene oxide (GO), an important derivative of graphene, contains abundant oxygen-containing functional groups, including hydroxyl, carboxyl, and epoxy groups, which impart hydrophilicity and amphiphilic characteristics to its surface and can substantially improve its compatibility with polymer matrices [19,20,21]. These characteristics make GO an attractive alternative to pristine graphene as a nanofiller for polymer composites. More importantly, the unique two-dimensional layered structure of GO enables the formation of an effective physical barrier within polymer matrices, thereby providing considerable potential for suppressing the migration of small molecules.

In the field of solid-propellant liners, Li et al. [22] pioneered the preparation of GO/HTPB composite liners and investigated their resistance to small-molecule migration. Their results demonstrated that the incorporation of GO significantly increased the crystallinity and crosslink density of the liner, while reducing the migration concentration of DOS by 23.28% and that of water molecules by 51.89%. Meanwhile, the bonding strength increased from 0.25 to 0.95 MPa. These findings provide important experimental evidence for the application of GO in anti-migration modification of propellant liners and establish a foundation for further development of GO-based liner materials. However, although the macroscopic barrier effect of GO has been demonstrated, the molecular-level relationship between GO incorporation and plasticizer migration remains insufficiently understood. In particular, the effects of GO on the thermodynamic interaction between DOS and the HTPB/IPDI matrix, and the corresponding molecular diffusion behavior require further clarification.

Although conventional experimental methods can provide valuable information on the migration behavior of plasticizers in solid propellants, they are generally associated with relatively long testing periods and certain safety concerns. More critically, conventional experimental approaches have considerable limitations in elucidating the key factors governing migration at the molecular level, such as intermolecular interactions, molecular diffusion pathways, and free volume. Therefore, experimental characterization alone is often insufficient to establish the intrinsic relationship between the molecular structure of liner materials and the migration behavior of plasticizers. Over the past several decades, computational simulation has emerged as a powerful tool for understanding complex physicochemical phenomena. Among various computational approaches, molecular dynamics (MD) simulation has been increasingly employed to investigate the compatibility of propellant components and the migration behavior of plasticizers [23]. One of the major advantages of MD simulation is its ability to independently control the parameters of a molecular system and establish well-defined thermodynamic states corresponding to experimental conditions. In MD simulations, a thermodynamic ensemble represents a collection of microscopic states that share the same macroscopic or thermodynamic properties. By selecting an appropriate ensemble, specific thermodynamic variables can be controlled while other variables are allowed to evolve during the simulation, enabling the molecular-level behavior of complex systems to be investigated under well-defined conditions [24].

Several studies have demonstrated the effectiveness of MD simulations in elucidating the migration behavior and interfacial interactions of energetic propellant systems. More recently, Fu et al. [25] combined MD simulations with experimental analysis to investigate the dynamic migration behavior of multiple components across the NEPE propellant/liner interface. Classical MD simulations involving energy minimization, thermodynamic equilibration, and dynamic equilibration were performed to characterize the migration of representative components, including NG and BTTN, from the propellant toward the liner. The mean square displacement, diffusion coefficient, and concentration-gradient distributions of these molecules were calculated to quantitatively evaluate their migration behavior. The results indicated that NG exhibited the highest diffusion coefficient, followed by BTTN, demonstrating the different migration tendencies of energetic plasticizer components within the propellant/liner system. Therefore, combining experimental migration measurements with MD simulations is expected to provide deeper insight into the molecular mechanism underlying the anti-migration effect of GO in HTPB-based liners [26,27].

In this work, GO was incorporated into an HTPB/IPDI liner to improve its resistance to DOS migration. Composite liners containing different GO contents (0, 0.1, 0.3, and 0.5 wt%) were prepared, and their anti-migration performance was systematically evaluated using immersion experiments. Molecular dynamics (MD) simulations were further employed to construct DOS/HTPB/IPDI and GO/DOS/HTPB/IPDI amorphous models, through which the binding energy and diffusion coefficient of DOS were calculated to elucidate the migration behavior at the molecular level. By combining experimental observations with molecular simulations, the relationship between GO incorporation, molecular interaction, diffusion behavior, and macroscopic anti-migration performance was established. This study provides molecular-level insight into the barrier effect of GO in HTPB-based liners and offers a practical strategy for the design of high-performance liner materials with enhanced resistance to plasticizer migration.

## 2. Experimental Section

### 2.1. Materials

Hydroxyl-terminated polybutadiene (HTPB, 99.9%, hydroxyl value ≥ 0.1 mol/100 g) was purchased from Tianjin Siense Biochemical Technology Co., Ltd., Tianjin, China. Isophorone diisocyanate (IPDI, ≥98.0%) was purchased from Shanghai Aladdin Company, Shanghai, China. Dioctyl Sebacate (DOS, ≥97.0%) was purchased from Shanghai Macleans Biochemical Technology Co., Ltd., Shanghai, China. Graphene oxide (GO) was prepared by a modified Hummers method; the corresponding characterization results have been provided in the Supporting Information (Figure S1).

### 2.2. Preparation of GO/HTPB Liner

**Figure 1:**
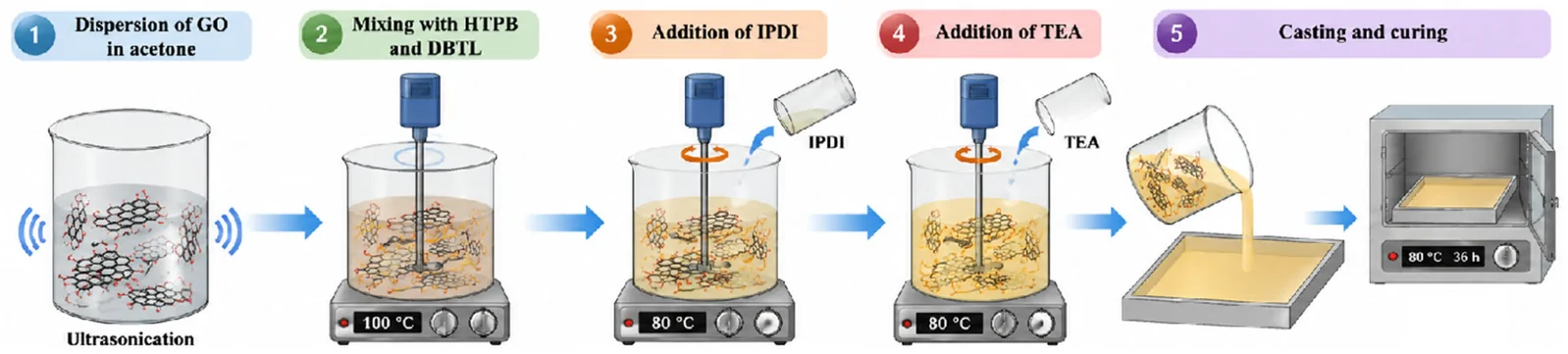
Preparation process of GO/HTPB composite liners.

In this experiment, GO/HTPB (GH) composite liners were prepared through the following five-step procedure (Figure 1). First, a predetermined amount of GO (0, 0.1, 0.3, or 0.5 wt%) was dispersed in acetone by ultrasonication to obtain a homogeneous GO suspension. Second, HTPB and dibutyltin dilaurate (DBTL) were added to the GO suspension at a mass ratio of 1:0.1% and mechanically stirred to ensure sufficient mixing. The resulting mixture was maintained at 100 °C to remove acetone completely, followed by the addition of IPDI according to a curing coefficient of *R* = 1.3. Third, the mixture was stirred in an oil bath at 80 °C for 2 h. Subsequently, triethylamine (TEA) was added and thoroughly mixed, after which the mixture was poured into a mold with dimensions of 150 × 150 × 2 mm. Finally, the mold was placed in an oven and cured at 80 °C for 36 h to obtain the GO/HTPB composite liners. After curing, the obtained HTPB/GO samples were designated as GH-0.1, GH-0.3, and GH-0.5 according to the GO content. The morphology of these samples was further characterized by scanning electron microscopy (SEM), and the corresponding images are provided in Figure S2 of the Supporting Information.

### 2.3. Characterization

Fourier-transform infrared (FTIR) spectra were obtained using a Nicolet IS-10 (Thermo Fisher Scientific, Waltham, MA, USA) spectrometer. Laser Raman spectra were measured on a Renishaw inVia spectrometer (Renishaw plc, Wotton-under-Edge, UK). The microstructure was examined by scanning electron microscopy (SEM, Hitachi S-4800; Hitachi High-Tech Corp., Tokyo, Japan). Soxhlet extraction was used to determine the density of crosslink of liners. The soluble component in the resin was extracted with acetone close to the boiling temperature, and the insoluble component was considered to be the cured resin. The cross-link density test process is shown in Figure S3.

### 2.4. Anti-Migration Performance Test by the Immersion Method

As shown in Figure S4, to determine the amount of DOS absorbed by the liner, the composite liners were cut into specimens with dimensions of 10 × 10 × 2 mm^3^, and their initial masses were recorded as m_0_. Each specimen was completely immersed in DOS, sealed, and placed in a constant-temperature oven at 30 °C. At predetermined time intervals, the specimen was removed from the DOS, and the excess DOS adhering to its surface was carefully removed before weighing. The specimen was then returned to the DOS for continued immersion. This procedure was repeated until the mass of the specimen remained essentially constant, indicating that the migration process had reached equilibrium. The mass of the specimen at time *t* was denoted as m_t_. Accordingly, the DOS concentration (C_t_) absorbed by the liner at time t was defined as the ratio of the mass increase to the current mass of the specimen:

(1) $$C_{t}=\frac{m_{t}-m_{0}}{m_{0}}\times 100\%$$

where *C_t_* represents the migration concentration of DOS, *m_t_* represents the mass of the diffused substance sheet including the absorbed substance, *m*_0_ represents the initial mass of sheet.

The immersion method was selected in this study to quantitatively evaluate the time-dependent DOS uptake and equilibrium migration behavior of the HTPB-based liner under controlled and identical conditions [10]. This approach enables direct comparison of the effect of GO incorporation on DOS migration and provides a macroscopic migration parameter that can be considered together with the molecular-scale diffusion behavior obtained from MD simulations. Although other approaches, including migration-cell and diffusion-cell methods, can also be employed to characterize plasticizer migration, they may involve different testing configurations and emphasize different aspects of the migration process. Since the objective of the present work was to compare the relative migration behavior of HTPB liners with different GO contents and establish a connection between experimental migration and molecular-scale transport, the immersion method was adopted as the primary experimental approach. To quantitatively evaluate the DOS transport kinetics, the experimentally measured DOS uptake data were further analyzed using Fick’s second law [28,29]. The experimental diffusion coefficient was obtained by fitting the time-dependent DOS uptake data with the Fickian diffusion model. The detailed fitting procedure and the corresponding equation are provided in the Supporting Information.

## 3. Molecular Dynamics Simulation

Molecular modeling and molecular dynamics simulations were performed using BIOVIA Materials Studio 2020 (Dassault Systèmes). Molecular structures of HTPB, IPDI, DOS, and GO were constructed using the Materials Visualizer module [30]. The HTPB polymer chain was constructed using the built-in Build Polymer functionality in Materials Studio. Geometry optimization and molecular dynamics simulations were performed using the Forcite module. The COMPASS force field was employed for all molecular modeling and simulation calculations, and atomic charges were assigned using the force-field charge assignment scheme.

The HTPB molecular chain consists of three types of butadiene units, including 11 cis-1,4 units, 11 1,2-vinyl units, and 33 trans-1,4 units, corresponding to an overall degree of polymerization of 55. The HTPB model was constructed by first drawing the cis-1,4-, trans-1,4-, and 1,2-vinyl butadiene monomer units using the Visualizer module. These monomer units were subsequently polymerized into a single molecular chain using the Build Polymer tool. Finally, the terminal carbon atoms of the molecular chain were modified with hydroxyl groups (–OH) to ensure hydroxyl termination of the HTPB chain.

To reduce size effects and avoid excessive computational costs, the molecular weight of the GO model was controlled below 5000 [31,32,33]. The GO structure was constructed according to a functional-group distribution of 0.5 carboxyl groups (–COOH), 2 hydroxyl groups (–OH), and 2 epoxy groups per 10 carbon atoms [23]. Specifically, a graphite crystal was first imported into Materials Studio, and a single-layer graphene sheet was cleaved along the (001) plane using the Cleave Surface tool. The lattice vectors *a* and *b* were then expanded by factors of 5 and 4, respectively, using the Build → Symmetry → SuperCell function, generating a graphene sheet containing 40 carbon atoms. The edges of the graphene sheet were saturated with hydrogen atoms. Subsequently, carboxyl groups were introduced at the edges, while hydroxyl and epoxy groups were randomly distributed on the graphene basal plane according to the specified functional-group ratio, yielding a GO model with a molecular weight below 5000.

**Figure 2:**
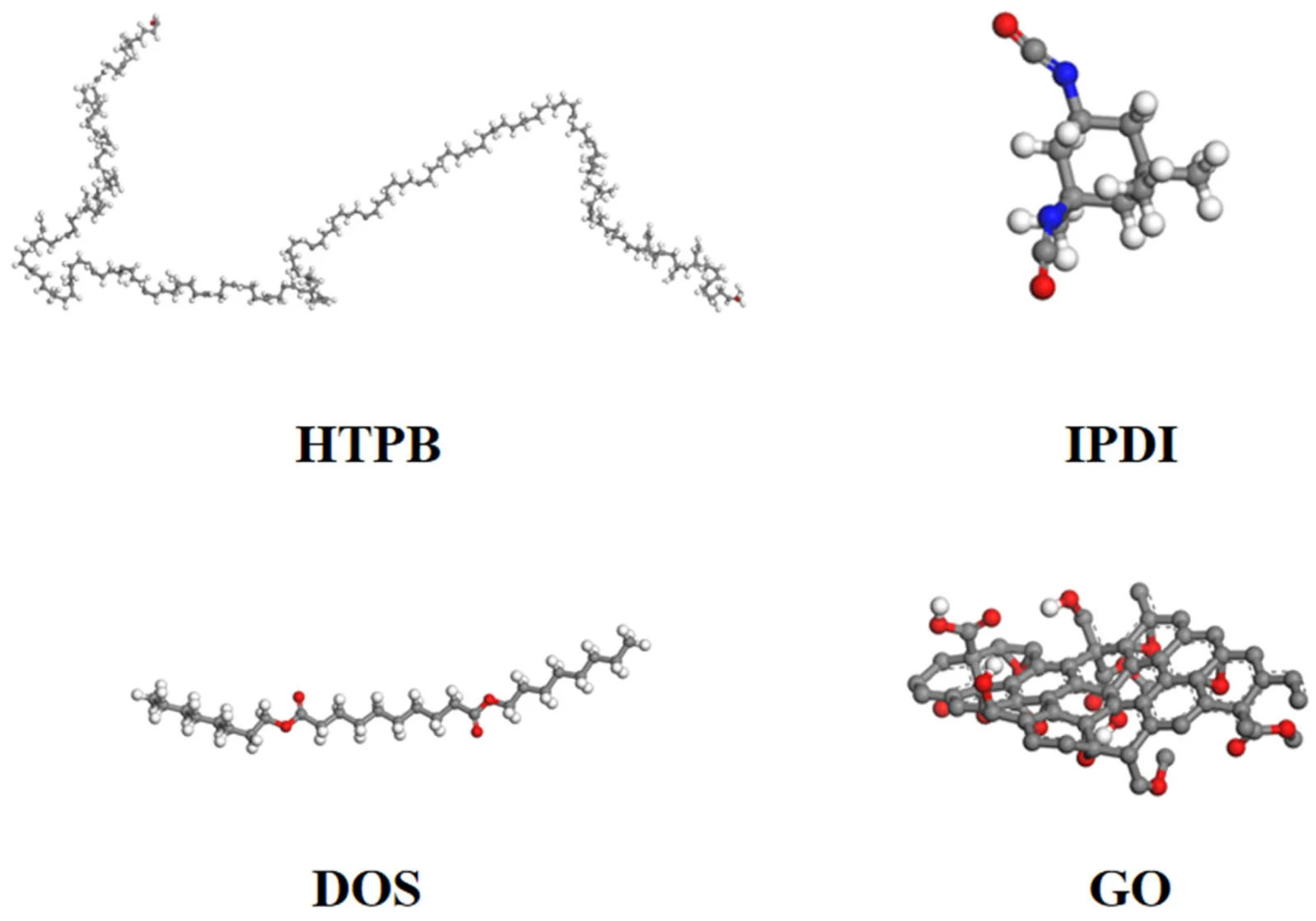
Molecular structures of HTPB, IPDI, DOS, and GO. The atom colors are defined as follows: gray, carbon (C); white, hydrogen (H); red, oxygen (O); and blue, nitrogen (N).

The geometric structures of GO, HTPB, IPDI, and DOS were subsequently optimized using the Geometry Optimization task in the Forcite module. The convergence criteria for energy and force were set to 1.0 × 10^−4^ kcal/mol and 0.005 kcal/mol/Å, respectively, while the pressure was set to 0.005 GPa. The COMPASS force field was employed for all calculations, with the calculation quality set to Fine and the atomic charges assigned using the Forcefield assigned option. Both electrostatic and van der Waals interactions were calculated using the Atom based summation method. The optimized molecular structures with the lowest-energy configurations were obtained and are presented in Figure 2.

Amorphous cell models of DOS/HTPB/IPDI and GO/DOS/HTPB/IPDI were constructed using the Construction task in the Amorphous Cell module. For the DOS/HTPB/IPDI model, the numbers of DOS, HTPB, and IPDI molecules were set to 5, 2, and 1, respectively. For the GO/DOS/HTPB/IPDI model, 5 DOS molecules, 1 GO molecule, 2 HTPB molecules, and 1 IPDI molecule were included. The COMPASS force field was employed, with atomic charges assigned using the Forcefield assigned option. The initial density and temperature of both amorphous cell models were set to 0.91 g/cm^3^ and 298.15 K, respectively. The Ewald summation method was employed to calculate electrostatic interactions with an accuracy of 1.0 × 10^−4^ kcal/mol. van der Waals interactions were calculated using the Atom based method, with a non-bonded cutoff distance of 9.5 Å, a spline width of 1 Å, and a buffer width of 0.5 Å.

The constructed DOS/HTPB/IPDI and GO/DOS/HTPB/IPDI amorphous cell models were subjected to energy minimization using the Geometry Optimization task in the Forcite module. Periodic boundary conditions (PBCs) were applied in all three Cartesian directions (x, y, and z) to represent a bulk condensed-phase environment and minimize finite-size and surface effects during the molecular dynamics simulations. The calculation parameters were kept consistent with those used for the constructing the amorphous cells. Following geometry optimization, molecular dynamics simulations were performed using the Dynamics task in the Forcite module, starting from the lowest-energy configurations of the two models. An NPT ensemble was employed at 298.15 K and 1.0 × 10^−4^ GPa, with a time step of 1.0 fs and a total simulation time of 200.0 ps, corresponding to 2.0 × 10^5^ simulation steps. The Nosé–Hoover thermostat and Berendsen barostat were selected for temperature and pressure control, respectively. The COMPASS force field and Forcefield assigned charge scheme were used throughout the simulations. Electrostatic interactions were treated using the Ewald summation method with an accuracy of 1.0 × 10^−4^ kcal/mol, while van der Waals interactions were calculated using the Atom based method with a cutoff distance of 9.5 Å.

**Figure 3:**
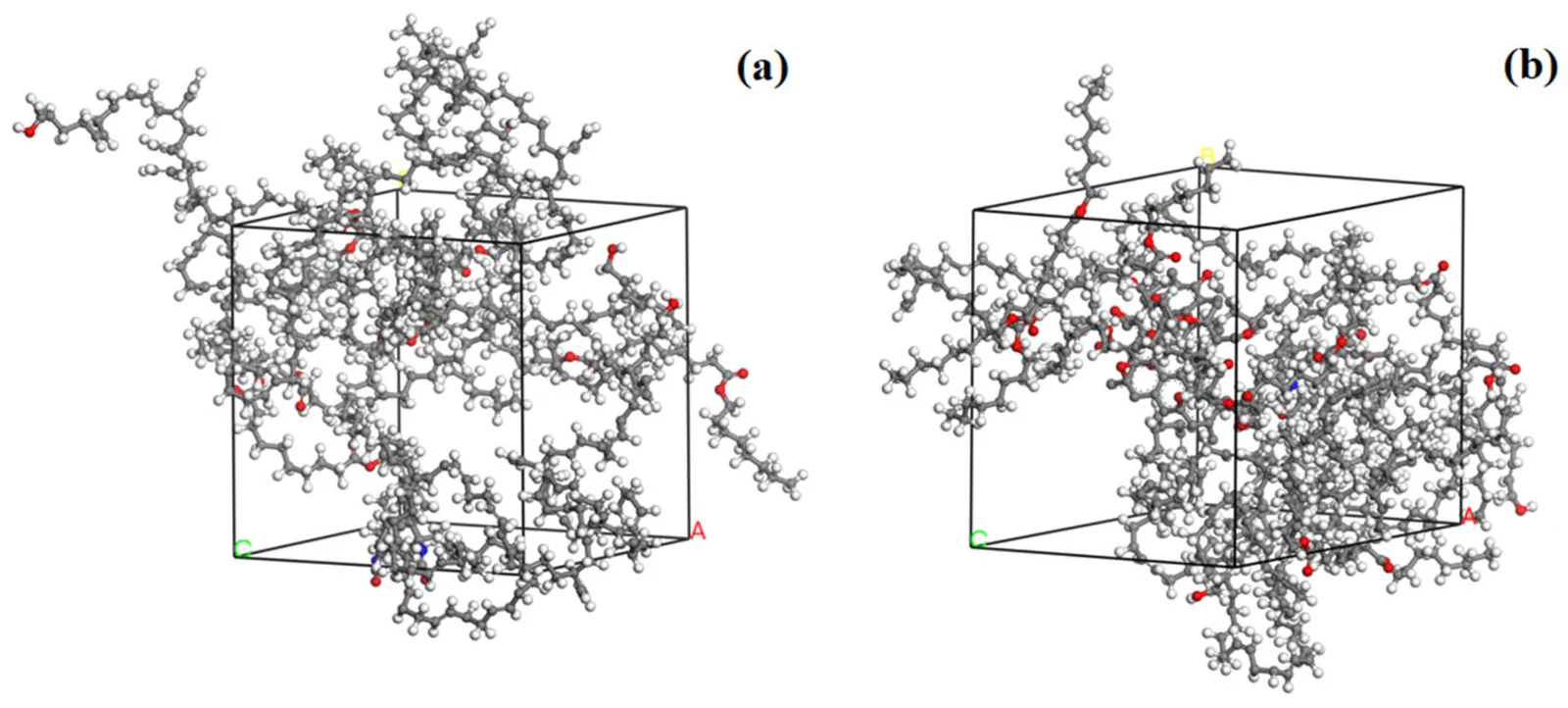
Molecular models of the blends (**a**) DOS/HTPB/IPDI, (**b**) GO/DOS/HTPB/IPDI.

After the MD simulations, thermodynamically equilibrated DOS/HTPB/IPDI and GO/DOS/HTPB/IPDI amorphous cell models were obtained, as shown in Figure 3. The corresponding MD trajectory files, which record the atomic configurations, atomic velocities, and other structural information at successive simulation steps, were subsequently used for the calculation and analysis of the relevant molecular-level parameters.

## 4. Results and Discussion

### 4.1. Anti-Plasticizer Migration Performance

**Figure 4:**
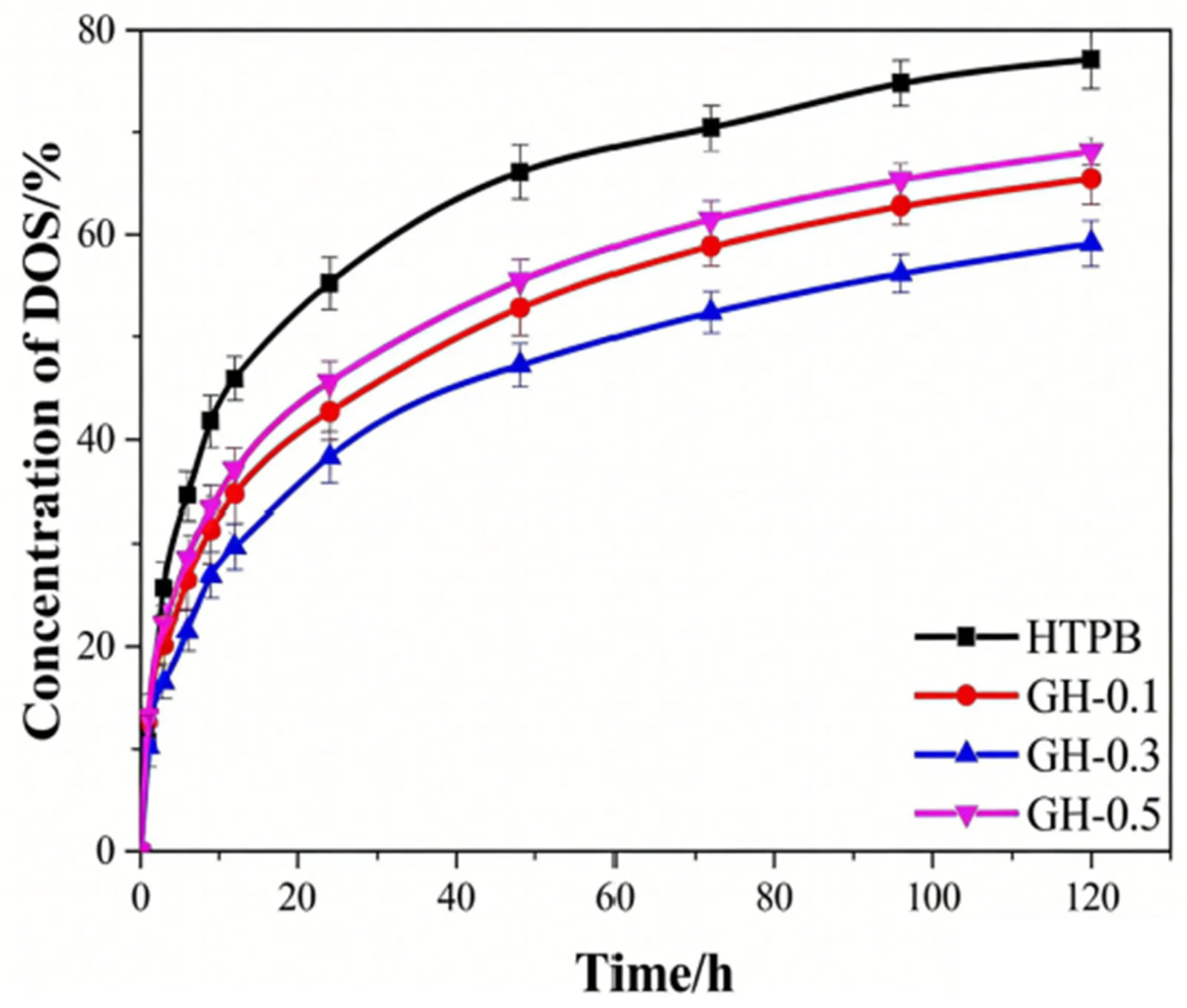
Curve of plasticizer migration amount in the liners changing with time at 25 °C.

Figure 4 presents the variation in DOS concentration with immersion time for GH composite liners containing different GO contents, as determined by the immersion method at 30 °C. Overall, the DOS concentration of all samples increased with increasing immersion time. However, the incorporation of GO significantly reduced the migration rate of DOS and consequently improved the anti-migration performance of the liner. Once migration reached equilibrium, the amount of DOS absorbed by the GH composite liners was considerably lower than that absorbed by the unmodified HTPB liner.

During the initial stage (0–20 h), the DOS concentration increased rapidly for all samples, corresponding to the rapid uptake of plasticizer near the liner surface. After 20 h, the DOS concentration of the neat HTPB liner continued to increase relatively rapidly, whereas the increase became progressively slower for the GO-containing samples. This behavior indicates that GO effectively hindered the diffusion of DOS within the HTPB matrix. The two-dimensional layered structure of GO can introduce additional barriers to molecular transport, thereby increasing the tortuosity of the diffusion pathways and reducing the rate at which DOS penetrates into the liner.

A comparison among the samples with different GO contents further revealed that GH-0.3 exhibited the lowest equilibrium DOS concentration, indicating the optimal anti-migration performance at a GO content of 0.3 wt%. The DOS concentration of GH-0.1 remained consistently lower than that of neat HTPB throughout the immersion period. Although GH-0.5 still exhibited better anti-migration performance than neat HTPB, its DOS concentration was slightly higher than that of GH-0.1 at most immersion times. This non-monotonic dependence on GO content suggests that an appropriate GO loading is critical for maximizing the barrier effect.

To quantitatively evaluate the effect of GO incorporation on DOS transport kinetics, the experimental DOS uptake data were further fitted using the Fickian diffusion model. The corresponding effective diffusion coefficients are summarized in Table S1 of the Supporting Information. The diffusion coefficient decreased from1.85 × 10^−7^ m^2^/s for GH-0 to 2.13 × 10^−8^, 2.13 × 10^−8^, and 2.78 × 10^−8^ m^2^/s for GH-0.1, GH-0.3, and GH-0.5, respectively. Thus, the incorporation of GO substantially reduced the effective diffusion coefficient of DOS, with GH-0.3 exhibiting the lowest value. These results are consistent with the equilibrium DOS uptake results and further demonstrate that an appropriate GO content can effectively suppress DOS transport.

To further clarify the effect of GO content on the structure of the cured HTPB matrix, the crosslinking degree of the GH liners was determined by the acetone extraction method, and the results are summarized in Table S2. The crosslinking degree increased from 90.43% for GH-0 to 95.58% for GH-0.1 and reached a maximum of 96.05% for GH-0.3, followed by a decrease to 94.06% for GH-0.5. The higher crosslinking degree at moderate GO contents indicates the formation of a relatively denser and more highly crosslinked polymer network, which can restrict chain mobility and reduce the available free volume for DOS transport. Notably, the highest crosslinking degree observed for GH-0.3 is consistent with its lowest equilibrium DOS concentration, suggesting that the denser crosslinked network contributes to the suppression of DOS transport.

Overall, the improved anti-migration performance at moderate GO contents can be attributed to the synergistic effects of the crosslinked polymer network and the physical barrier effect of well-dispersed GO nanosheets. GO nanosheets can increase the tortuosity of the diffusion pathways, while the relatively high crosslinking degree restricts polymer-chain mobility and molecular transport. At excessive GO loading, however, possible local aggregation and the associated decrease in crosslinking degree may reduce the uniformity and continuity of the barrier network. Therefore, an optimal GO content is required to achieve a balance between effective barrier formation, crosslinking, and filler dispersion.

### 4.2. MD Simulation of Plasticizer Migration

In MD simulations, the attainment of thermodynamic equilibrium can generally be evaluated based on the evolution of temperature and energy-related parameters, including the total energy, potential energy, kinetic energy, and non-bonded interaction energy [34,35,36]. When these parameters become stable with time or fluctuate within an acceptable range of approximately 5–10%, the simulated system can be considered to have reached thermodynamic equilibrium.

**Figure 5:**
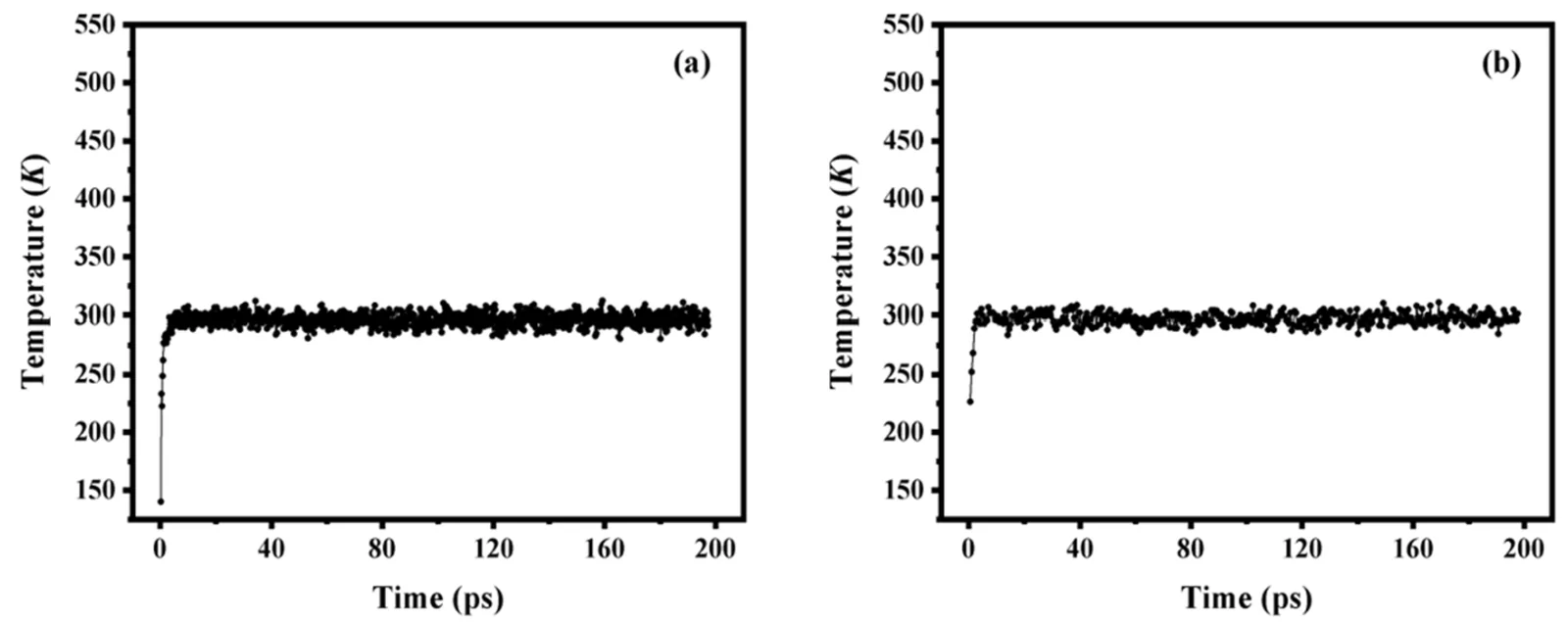
Temperature variation with time for the DOS/HTPB/IPDI blend system (**a**) and the GO/DOS/HTPB/IPDI blend system (**b**).

**Figure 6:**
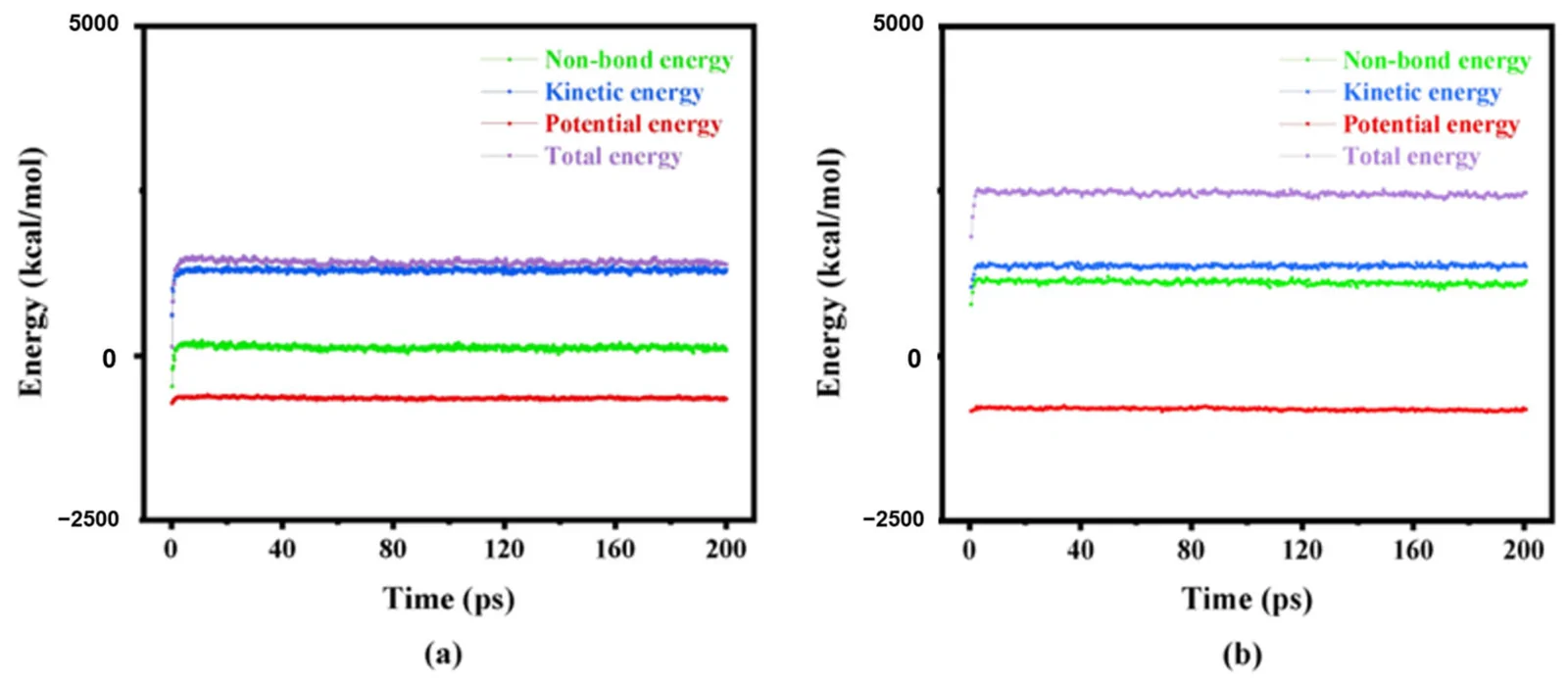
Time-dependent curves of the total energy, kinetic energy, potential energy, and non-bonded interaction energy for the DOS/HTPB/IPDI blend model (**a**) and the GO/DOS/HTPB/IPDI blend model (**b**).

Figure 5 shows the temperature evolution of the DOS/HTPB/IPDI (a) and GO/DOS/HTPB/IPDI (b) systems, while Figure 6 presents the variations in total energy, kinetic energy, potential energy, and non-bonded interaction energy for the two systems. As shown in Figure 5, noticeable fluctuations in temperature and energy-related parameters are observed during the initial stage of the simulations, particularly before approximately 50 ps. This behavior can be attributed to the relaxation of the initial molecular configurations toward their equilibrium states [37,38,39,40,41]. After 50 ps, the temperatures of both systems remain stable around the target temperature of 298 K, with fluctuations within approximately ±10 K. Meanwhile, the individual energy components gradually reach stable values without obvious systematic drift or pronounced oscillations.

These results indicate that both the DOS/HTPB/IPDI and GO/DOS/HTPB/IPDI systems have reached thermodynamic equilibrium after approximately 50 ps of simulation. Therefore, the trajectories obtained during the equilibrated stage can be extracted for subsequent calculations of the binding energy and diffusion coefficient of DOS, providing a reliable molecular basis for analyzing the effect of GO on the migration behavior of DOS.

#### 4.2.1. Binding Energy Analysis

Generally, the compatibility between different materials essentially depends on the strength of intermolecular interactions. When the intermolecular interaction of different materials is strong, the closer the interfacial bonding is, the better the compatibility is. E_binding_ is used to evaluate the compatibility of the two components in the blends. It is defined as the negative value of intermolecular interaction energy (*E*_inter_). The intermolecular interaction energy is calculated based on the total energy of the blend and the energy of each component under equilibrium conditions. Taking DOS/HTPB/IPDI blends as an example, *E*_binding_ is calculated according to the Formula (2) [42]:

*E*_binding_ = −(*E*_total_ − *E*_HTPB/IPDI_ − *E*_DOS_) (2)

where *E_total_* is the total energy of the mixture, E*_HTPB/IPDI_* and E*_DOS_* are the total energy of HTPB/IPDI and DOS in equilibrium state, respectively.

**Figure 7:**
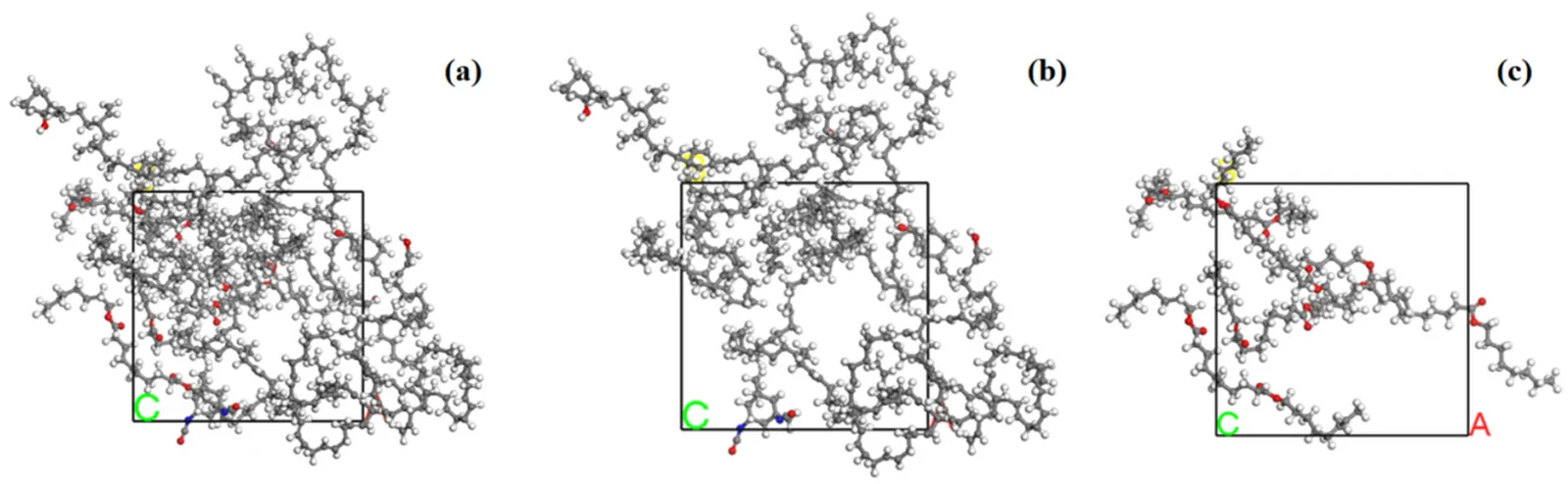
Molecular models of (**a**) the DOS/HTPB/IPDI blend, (**b**) the HTPB/IPDI blend, and (**c**) DOS.

**Figure 8:**
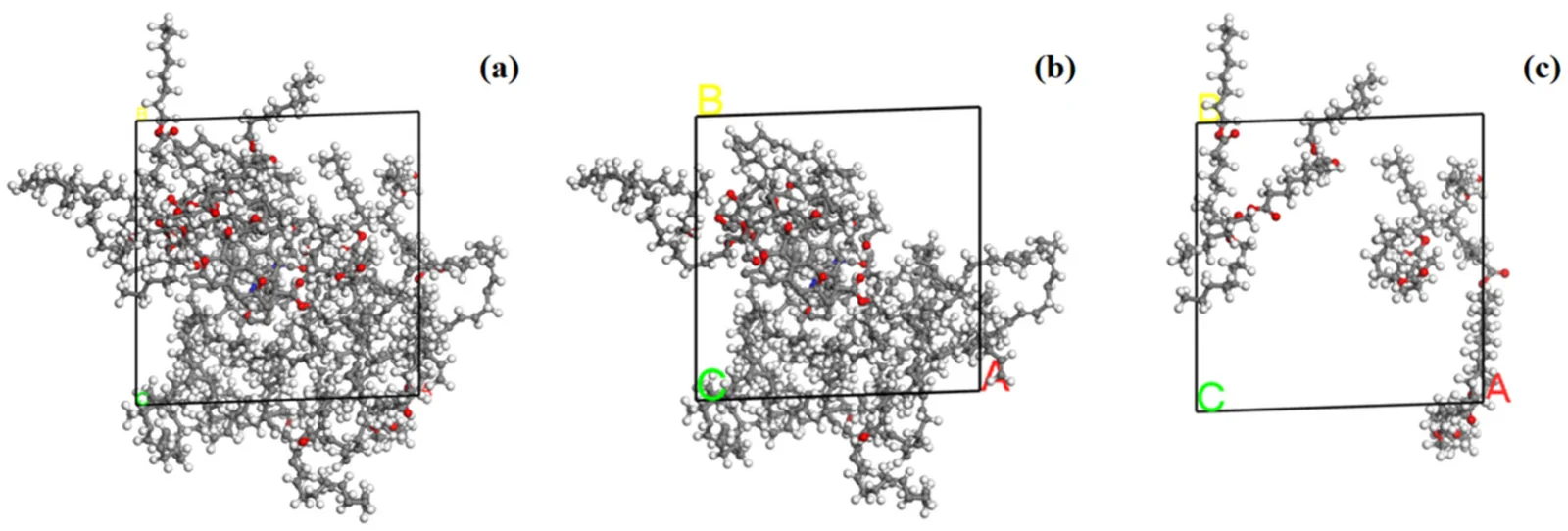
Molecular models of (**a**) the DOS/GO/HTPB/IPDI blend, (**b**) the GO/HTPB/IPDI blend, and (**c**) DOS.

As shown in Figure 7 and Figure 8, the binding energy values summarized in Table 1 and Table 2 reveal a clear difference in the intermolecular interactions between DOS and the matrix. The binding energy of DOS with the HTPB/IPDI matrix is 290.998 kcal/mol, whereas that of DOS with the GO/HTPB/IPDI matrix decreases to 229.2641 kcal/mol. The substantially lower binding energy in the GO-containing system indicates that the interaction between DOS and the composite matrix is significantly weakened after the incorporation of GO.

This reduction in binding energy suggests that GO alters the molecular interaction environment within the HTPB/IPDI matrix and decreases the affinity of DOS toward the matrix. In other words, the incorporation of GO introduces an additional barrier within the polymer network, which reduces the favorable interaction between DOS molecules and the surrounding matrix and consequently restricts their penetration into the liner. Therefore, the binding energy results provide molecular-level evidence for the barrier effect of GO and support the experimentally observed suppression of DOS migration. These findings also provide a molecular basis for the subsequent analysis of the DOS diffusion coefficient.

#### 4.2.2. Migration Coefficient

The diffusion coefficient (*D*), also referred to as the migration coefficient, is a fundamental kinetic parameter describing the migration rate of small molecules within a polymer matrix [43,44,45]. For the plasticizer dioctyl sebacate investigated in this study, a larger D indicates that DOS molecules can travel a greater distance within a given period, corresponding to stronger migration ability and poorer anti-migration performance. Conversely, a lower *D* indicates restricted molecular diffusion and, consequently, enhanced resistance to plasticizer migration.

In molecular dynamics simulations, the *D* can be calculated from the mean square displacement (MSD) using the Einstein relation [46]. If the positions of the ith DOS molecule at time t and the initial time are denoted as r_i_(t) and r_i_(0), respectively, the MSD is defined as the ensemble average of the squared displacement of the DOS molecules over time:

(3) $$MSD=s\left(t\right)=\langle {\left|r\left(t\right)-r\left(0\right)\right|}^{2}\rangle$$

where the angular brackets denote averaging over the DOS molecules and different initial time origins. For random diffusion in a three-dimensional space, the Einstein relation describes the relationship between the diffusion coefficient *D* and MSD as:

(4) $$D=\underset{t\to \infty }{lim}\frac{\langle \;|r\left(t\right)-r\left(0\right){|}^{2}\rangle }{6t}$$

Once the system has reached thermodynamic equilibrium, the MSD generally exhibits an approximately linear increase over a sufficiently long interval. Therefore, the linear region of the MSD–time curve can be fitted using the least-squares method to obtain the slope *m* [47,48,49]. According to the Einstein relation, the diffusion coefficient of DOS can then be expressed as:

*D* = *s(t)*/6t = m/6 (5)

where m is the slope of the linear region of the MSD–time curve. The diffusion coefficient obtained from the MD trajectories can therefore be used to quantitatively compare the migration behavior of DOS in the HTPB/IPDI and GO/HTPB/IPDI systems. A lower D value indicates more restricted molecular diffusion and a stronger barrier effect of GO.

To maintain consistency with the MD calculation results, the diffusion coefficients are reported in m^2^/s after conversion from the MSD–time units used in the simulation. This analysis provides a quantitative molecular-level basis for evaluating the effect of GO incorporation on the migration kinetics of DOS.

**Figure 9:**
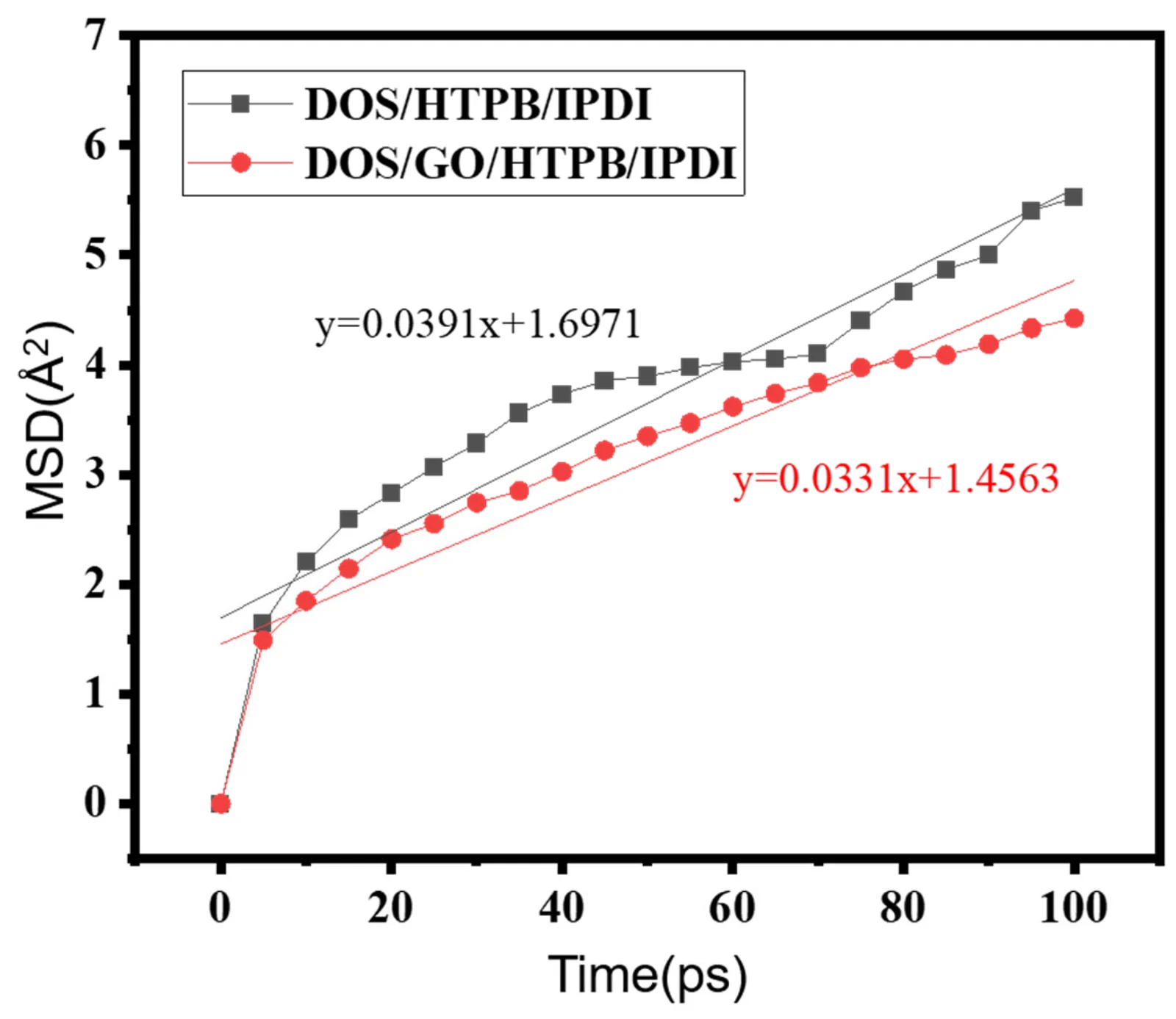
Curves of MSD of plasticizer migration.

Figure 9 presents the MSD curves of DOS as a function of simulation time for the DOS/HTPB/IPDI and DOS/GO/HTPB/IPDI systems. As shown in Figure 8, the MSD curve of the GO-free DOS/HTPB/IPDI system exhibits a relatively larger slope. Linear fitting of the MSD data gives a slope of *k*_1_, and the corresponding diffusion coefficient of DOS is calculated to be approximately 6.52 × 10^−11^ m^2^/s according to *D* = *k*_1_/6. In contrast, the MSD curve of the GO-containing DOS/GO/HTPB/IPDI system flattens progressively, yielding a smaller fitted slope (*k*_2_) and a diffusion coefficient of approximately 5.52 × 10^−11^ m^2^/s. Compared with the GO-free system, the incorporation of GO decreases the diffusion coefficient of DOS by approximately 15.3%. Although the diffusion coefficients of the two systems remain within the same order of magnitude, their time-dependent diffusion behaviors exhibit an evident difference.

During the initial simulation stage (0–50 ps), the MSD curves of the two systems are relatively close, indicating similar initial DOS diffusion behavior in both matrices. As the simulation proceeds, however, the MSD of DOS in the GO-free system continues its relatively steep ascent, whereas the slope of the MSD curve in the GO-containing system gradually decreases. Consequently, the difference between the two systems becomes increasingly pronounced at longer simulation times, indicating the barrier effect of GO intensifies over extended diffusion times and effectively retards the long-term migration of DOS. Such behavior is particularly relevant to the long-term storage of solid rocket motors, during which the cumulative migration of plasticizer can gradually impair the integrity and stability of the liner/propellant interface.

Integrating the experimental results with the MD simulations, the incorporation of GO suppresses DOS migration primarily through two complementary effects. First, the GO nanosheets are dispersed within the HTPB/IPDI matrix and provide a two-dimensional physical barrier [50,51,52,53]. Because DOS molecules cannot readily penetrate the graphene oxide nanosheets, their diffusion is forced to proceed around the nanosheets, resulting in a more tortuous diffusion pathway and a corresponding reduction in the effective migration rate. This interpretation is directly supported by the MD results, where the slope of the MSD curve for the GO-containing system is lower than that of the GO-free system, with the calculated diffusion coefficient decreasing from 6.52 × 10^−11^ to 5.52 × 10^−11^m^2^/s, corresponding to a reduction of approximately 15.3%.

Second, GO modifies the intermolecular interaction environment of the HTPB/IPDI matrix and reduces the affinity between DOS and the surrounding matrix [54,55,56,57]. The binding energy decreases from 290.998 to 229.264 kcal/mol after the incorporation of GO, indicating weakened DOS–matrix interactions. This reduced interaction reflects the altered molecular environment introduced by GO and suggests that DOS is less strongly associated with the polymer matrix. Importantly, this reduced affinity does not result in an acceleration of DOS migration in the present system. Instead, under the constraint of the GO-induced physical barrier network, the weakened DOS–matrix interaction acts together with the increased diffusion tortuosity to limit the overall transport of DOS through the composite liner.

Therefore, the enhanced anti-migration performance of the GO-modified liner can be attributed to the synergistic effect of the two-dimensional GO barrier and the modified DOS–matrix interactions. The former primarily increases the effective diffusion path and imposes kinetic resistance to molecular transport, while the latter changes the molecular affinity and transport environment of DOS. The combined effects lead to a lower diffusion coefficient and ultimately provide the GO/HTPB liner with improved resistance to long-term DOS migration.

### 4.3. Comparison Between Experimental and MD Results

The experimental and molecular dynamics results show a consistent trend in the effect of GO on DOS migration. The immersion experiments demonstrated that GO incorporation decreased the DOS uptake of the HTPB liner, with GH-0.3 exhibiting the lowest equilibrium DOS concentration among the investigated formulations. In parallel, the MD simulations revealed that the diffusion coefficient of DOS decreased from 6.52 × 10^−11^ to 5.52 × 10^−11^ m^2^/s after GO incorporation, corresponding to a reduction of approximately 15.3%. The reduced slope of the MSD curve further indicates restricted long-time diffusion of DOS in the GO-containing system. The experimental Fickian analysis and MD simulations consistently demonstrate that GO incorporation suppresses DOS transport.

It should be noted that the experimental equilibrium DOS concentration and the MD-derived diffusion coefficient describe different aspects of the migration process and therefore cannot be directly compared in terms of their absolute numerical values. The experimental results characterize the macroscopic uptake behavior of DOS, whereas the diffusion coefficient obtained from MD simulations quantitatively describes the molecular-scale transport kinetics within the simulated HTPB/IPDI matrix. Accordingly, the MD calculations are primarily used to provide molecular-level mechanistic insight into the experimentally observed migration behavior rather than to directly reproduce the experimental equilibrium concentration.

## 5. Conclusions

In this study, the effect of GO incorporation on the anti-migration performance of HTPB-based liners was systematically investigated by combining experimental measurements with molecular dynamics simulations. The migration experiments demonstrated that the incorporation of GO significantly improved the resistance of the HTPB liner to plasticizer migration. Compared with the unmodified HTPB liner, the composite containing 0.3 wt% GO exhibited the lowest equilibrium concentration of DOS and therefore the optimal anti-migration performance.

The molecular dynamics simulations further demonstrated that GO incorporation decreased the diffusion coefficient of DOS from 6.52 × 10^−11^ to 5.52 × 10^−11^ m^2^/s, corresponding to a reduction of approximately 15.3%. The MSD curves also showed a significantly lower diffusion rate of DOS in the GO-containing system, confirming that GO can effectively retard the migration of DOS molecules. Binding energy analysis revealed that the binding energy between DOS and the HTPB/IPDI matrix decreased from 290.998 to 229.264 kcal/mol after GO incorporation, indicating reduced affinity between DOS and the polymer matrix and thereby contributing to the suppression of plasticizer migration.

Overall, the two-dimensional layered structure of GO forms an effective physical barrier within the HTPB matrix, forcing DOS molecules to follow more tortuous diffusion pathways and thereby increasing the resistance to molecular transport. This GO-induced barrier effect, together with the modified DOS–matrix interactions, constitutes the primary mechanism responsible for the enhanced anti-migration performance of the GO/HTPB liner. These findings demonstrate the potential of GO as an effective nanofiller for developing HTPB-based liner materials with improved resistance to plasticizer migration.

## Figures and Tables

**Table 1:** Binding energy between DOS and the HTPB/IPDI matrix.

*E*_total_ (kcal/mol)	*E*_DOS_ (kcal/mol)	*E*_HTPB/IPDI_ (kcal/mol)	*E*_binding_ (kcal/mol)
79.151902	33.354046	336.795859	290.998

**Table 2:** Binding energy between DOS and the GO/HTPB/IPDI matrix.

*E*_total_ (kcal/mol)	*E*_DOS_ (kcal/mol)	*E*_GO/HTPB/IPDI_ (kcal/mol)	*E*_binding_ (kcal/mol)
1015.310357	29.702581	1214.871915	229.2641

## Data Availability

The original contributions presented in this study are included in the Supplementary Material. Further inquiries can be directed to the corresponding authors.

## Supplementary Materials

The following supporting information can be downloaded at: https://www.mdpi.com/article/10.3390/polym18202490/s1, Figure S1. FT-IR spectrum(a) and Raman spectrum(b) of GO; Figure S2. SEM images of the fracture surface of GH composite liners with different GO loadings; Figure S3. Flow chart of cross-link density test; Figure S4. Schematic illustration of the immersion test for evaluating the anti-migration performance of the GO/HTPB composite liners; Table S1. Migration coefficient of DOS in GH liners and linear correlation coefficient; Table S2. The calculated crosslinking degree of the GH liners.
