# Supplementary material for: Graphene Oxide-Modified Hydroxy-Terminated Polybutadiene Liner for Suppressing Plasticizer Migration: Molecular Dynamics Simulation and Experimental Study
Source: Polymers (Basel). 2026 Oct 12;18(20):2490. doi: 10.3390/polym18202490 (PMC13709169; doi:10.3390/polym18202490)
Supplement: Supplementary file 1 [file polymers-18-02490-s001.zip › polymers-4585280-supplementary.pdf]

Supporting Information

Graphene Oxide-Modified Hydroxy-Terminated Polybutadiene Liner for Suppressing Plasticizer Migration: Molecular Dynamics Simulation and Experimental Study

Junqiang Feng 1,2, Wanru Cheng 1, Liang Xu 3, Yuhan Meng 1, Chenxing Liu 3, Tao Ding 1, Yubing Hu 4 , Jun Zhou 5,*, Fuyao Chen 6 and Zhehong Lu 1,*

[1] College of Chemistry and Molecular Sciences, Henan University, Kaifeng 475004, China.

[2] Shanxi Jiangyang Chemical Co., Ltd., Taiyuan 030041, China

[3] Queen’s Business School, Queen's University Belfast, Belfast BT9 5AH, UK

[4] School of Chemistry and Chemical Engineering, Nanjing University of Science and Technology, Nanjing 210094, China

[5] School of Physics and Materials Engineering, Hefei Normal University, Hefei 230061, P. R. China

[6] Zhejiang Sci-Tech University Tongxiang Research, Jiaxing 314000, China

* Correspondence: E-mail: zhouj1222@126.com (Jun Zhou); luzhehong@henu.edu.cn(Zhehong Lu)

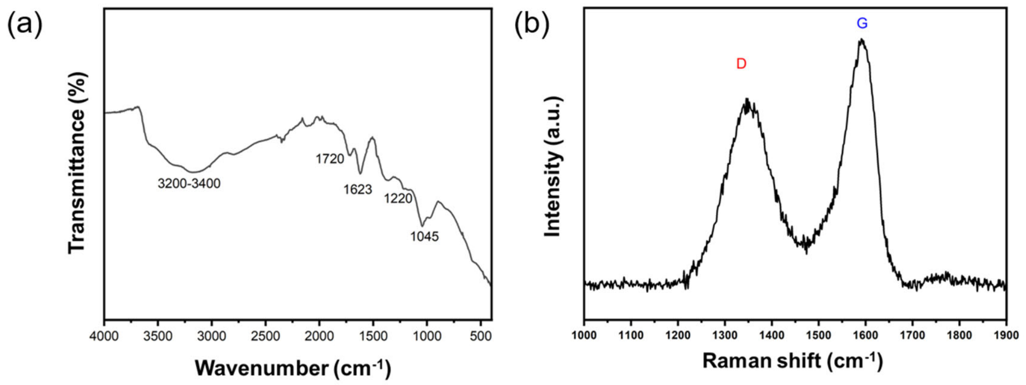

Figure S1. FT-IR spectrum(a) and Raman spectrum(b) of GO.

Figure S1(a) shows the infrared spectrum of GO. As can be seen from the figure, GO exhibits a broad and strong absorption peak at 3200-3400 cm^{-1}, which is the stretching vibration peak of hydroxyl groups. This is attributed to the presence of -OH functional groups on the surface of graphite after oxidation, as well as the vibration of water adsorbed by GO in the infrared spectrum. The absorption peak at 1720 cm^{-1} corresponds to the stretching vibration of C=O, the absorption peak at 1226 cm^{-1} corresponds to the stretching vibration of C-O, the absorption peak at 1045 cm^{-1} corresponds to the stretching vibration of C-O-C, and the absorption peak at 1623 cm^{-1} is the deformation vibration peak of adsorbed water molecules. The above results indicate that GO contains oxygen-containing functional groups such as hydroxyl, carboxyl, and epoxy groups. Figure S2(b) shows the typical Raman spectrum of GO obtained at an excitation wavelength of 532 nm. The main characteristics of the Raman spectrum of carbon-based materials are the D peak and the G peak. It can be observed that the D peak, which stands out at 1348 cm^{-1}, has a slightly lower intensity than the G peak at 1592 cm^{-1}, and their bandwidths are relatively large, indicating that there is significant structural disorder in GO.

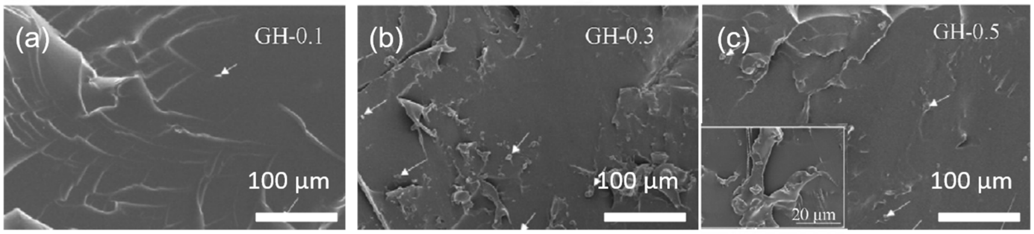

Figure S2. SEM images of the fracture surface of GH composite liners with different

GO loadings.

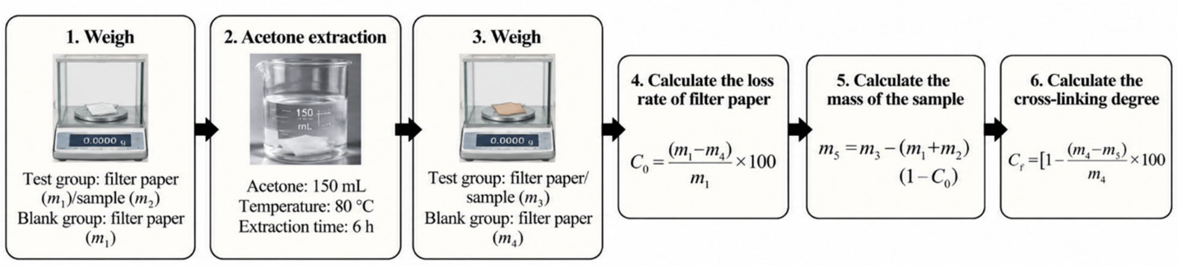

Figure S3. Flow chart of cross-link density test.

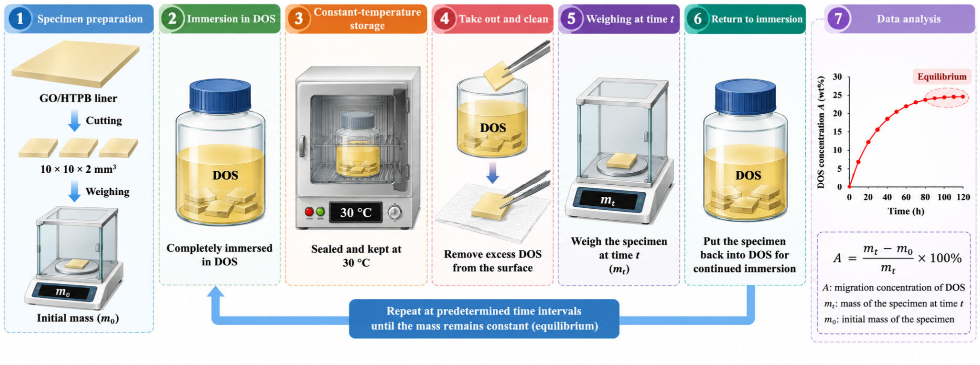

Figure S4. Schematic illustration of the immersion test for evaluating the anti-migration performance of the GO/HTPB composite liners.

TableS1. Migration coefficient of DOS in GH liners and linear correlation coefficient.

Samples	diffusion coefficient(m^2/s)	R^2
GH-0	1.85*10^{-7}	0.993
GH-0.1	2.13*10^{-8}	0.997
GH-0.3	1.31*10^{-8}	0.992
GH-0.5	2.78*10^{-8}	0.999

TableS2. The calculated crosslinking degree of the GH liners.

Samples	crosslinking degree (%)
GH-0	90.43
GH-0.1	95.58
GH-0.3	96.05
GH-0.5	94.06

Migration kinetics

As an important migration kinetic parameter, the migration coefficient can vividly describe the speed of the migration process, and the migration data is further processed to obtain the migration coefficient. According to Fick's second law:

$$\frac{\partial c}{\partial t} = D\frac{\partial^2 c}{\partial x^2}$$

Assuming that the migration is unidirectional under non-steady state conditions, with a constant diffusion coefficient, the amount of migration through the sample:

$$\frac{M_t}{M_\infty} = 1 - \frac{8}{\pi^2}\sum_{n=0}^{+\infty}\frac{1}{(2n+1)^2}exp\left[-\frac{D(2n+1)^2\pi^2}{{d_i}^2}t\right]$$

Simplified:

$$\frac{M_t}{M_\infty} = \frac{4}{d_i}\left(\frac{Dt}{\pi}\right)^{1/2}$$

Where M_t is the amount of migration at time t, and M_∞ is the amount of migration at equilibrium.

List the expression formula of the required data and convert it into the existing parameter form:

$$m_0=\rho_i d_i A，M_\infty=\frac{1}{2}\rho d_i At(x)，M_t=\Delta m=\frac{4}{d}t^{1/2}\left(\frac{D}{\pi}\right)^{1/2}\cdot M_\infty$$

$$\frac{\frac{\Delta m}{m_0}}{\left(t^{\frac{1}{2}}\right)} = \frac{2\rho t(x)}{\rho_i d_i}\left(\frac{D}{\pi}\right)^{\frac{1}{2}}$$

Derivative of the differential to obtain the calculation formula of the migration coefficient [3]:

$$\frac{d}{d\sqrt{t}}\left(\frac{\Delta m}{m_0}\right) = \frac{2\rho}{\rho_i d_i}\left(\frac{D}{\pi}\right)^{1/2}$$

Where Δm is the amount of DOS migrated into the liner at time t, m0 is the initial mass of the liner, ρ is the density of DOS 0.918g/cm^3, ρi is the liner density, and di is the thickness of the liner. Use t1/2 and Δm/m_0 as the abscissa and ordinate respectively to draw the point diagram, and the linear slope obtained after linear fitting can be calculated by the formula of Fick's second law to calculate the migration coefficient.
